# Protein tyrosine phosphatase-1B (PTP1B) helps regulate EGF-induced stimulation of S-phase entry in human corneal endothelial cells

**Published:** 2008-01-16

**Authors:** Yutaka Ishino, Cheng Zhu, Deshea L. Harris, Nancy C. Joyce

**Affiliations:** 1 Schepens Eye Research Institute, Boston, MA; 2 Department of Ophthalmology, Kyoto Prefectural University of Medicine, Kyoto, Japan; 3 Department of Ophthalmology, Harvard Medical School, Boston, MA

## Abstract

**Purpose:**

Human corneal endothelial cells (HCEC), particularly from older donors, only proliferate weakly in response to EGF. The protein tyrosine phosphatase, PTP1B, is known to negatively regulate EGF-induced signaling in several cell types by dephosphorylating the epidermal growth factor receptor (EGFR). The current studies were conducted to determine whether PTP1B plays a role in regulating cell cycle entry in HCEC in response to EGF stimulation.

**Methods:**

Donor corneas were obtained from the National Disease Research Interchange and accepted for study based on established exclusion criteria. PTP1B was localized in the endothelium of ex vivo corneas and in cultured cells by immunocytochemistry. Western blot analysis verified PTP1B protein expression in HCEC and then compared the relative expression of EGFR and PTP1B in HCEC from young (<3 years old) and older donors (>60 years old). The effect of inhibiting the activity of PTP1B on S-phase entry was tested by comparing time-dependent BrdU incorporation in subconfluent HCEC incubated in the presence or absence of the PTP1B inhibitor, CinnGEL 2Me, before EGF stimulation.

**Results:**

PTP1B was localized in a punctate pattern mainly within the cytoplasm of HCEC in ex vivo corneas and cultured cells. Western blots revealed the presence of three PTP1B-positive bands in HCEC and the control. Further western blot analysis showed no significant age-related difference in expression of EGFR (p=0.444>0.05); however, PTP1B expression was significantly higher in HCEC from older donors (p=0.024<0.05). Pre-incubation of HCEC with the PTP1B inhibitor significantly increased (p*=*0.019<0.05) the number of BrdU positive cells by 48 h after EGF stimulation.

**Conclusions:**

Both immunolocalization and western blot studies confirmed that PTP1B is expressed in HCEC. Staining patterns strongly suggest that at least a subset of PTP1B is localized to the cytoplasm and most likely to the endoplasmic reticulum, the known site of EGFR/PTP1B interaction following EGF stimulation. PTP1B expression, but not EGFR expression, was elevated in HCEC from older donors, suggesting that the reduced proliferative activity of these cells in response to EGF is due, at least in part, to increased PTP1B activity. The fact that inhibition of PTP1B increased the relative number of cells entering S-phase strongly suggests that PTP1B helps negatively regulate EGF-stimulated cell cycle entry in HCEC. These results also suggest that it may be possible to increase the proliferative activity of HCEC, particularly in cells from older donors, by inhibiting the activity of this important protein tyrosine phosphatase.

## Introduction

Human corneal endothelial cells (HCEC) do not normally proliferate in vivo [1,2]; however, they are able to divide in response to wounding in ex vivo corneas [3] and will proliferate in culture [4,5] when stimulated by appropriate growth promoting agents. Studies exploring the relative proliferative capacity of HCEC have demonstrated that HCEC from older donors (>50 years old) are less responsive to mitogens than cells from younger donors (<30 years old) [3-6]. For example, epidermal growth factor (EGF) is able to stimulate proliferation in HCEC [5-9], but the relative level of DNA synthesis and the number of cells that divide in response to EGF stimulation is relatively low, particularly in cells from older donors [5]. Studies are being conducted in this laboratory to identify the molecular basis for this weak response to determine whether it is possible to increase the sensitivity of HCEC to EGF. This could lead to development of methods to increase proliferation of HCEC in vivo or in donor corneas to be used for transplantation, resulting in increased endothelial cell density. It may also improve methods for use of HCEC in regenerative therapies.

Several growth factors, including EGF, signal via membrane-bound receptors with intrinsic protein-tyrosine kinase activity. Receptors of this type are termed receptor tyrosine kinases (RTKs). In the case of the EGF receptor (EGFR), the RTK activity is stimulated by ligand binding, resulting in autophosphorylation of specific tyrosine residues (Tyr992 and Tyr1148), which are located within the COOH-terminal intracellular domain of the receptor [10]. This tyrosine phosphorylation is reversible and plays an important role in activating and regulating downstream cellular pathways. Downstream mediators of EGF-induced signaling include phospholipase C-γ (PLC-γ) and its downstream calcium- and protein kinase C (PKC) cascades, as well as MAP kinases, including ERK/MAPK [10-12]. After ligand binding and tyrosine autophosphorylation, EGFR is rapidly internalized into endosomes and remains active for several minutes before either being sorted to lysosomes for degradation or recycled back to the plasma membrane. The fate of the receptor and the output of the signaling process depend on continued ligand binding and kinase activity [13,14].

Protein tyrosine phosphatases (PTPs) comprise a large family of receptor-like and non-receptor enzymes that share a highly conserved catalytic domain specific for phosphotyrosine hydrolysis. PTPs act as “on” and “off” switches for numerous signaling events, thus acting as regulators of the signaling process [15,16]. PTP1B is a widely expressed non-receptor PTP that was originally identified in placenta [17]. Sequencing studies indicate that PTP1B consists of 435 amino acids with a molecular weight of approximately 50 kDa [18-20]. PTP1B contains a phosphatase catalytic domain at its NH_2_-terminus (residues 40–276), a region containing proline-rich motifs that promote interaction with proteins containing SH_2_-domains. PTP1B also contains a COOH-terminal hydrophobic region (residues 401–431) that is necessary and sufficient to localize PTP1B to the cytoplasmic face of the endoplasmic reticulum [18,20-22] and nuclear envelope [23]. PTP1B interacts with and dephosphorylates several RTKs, including EGFR [24-26], the PDGF-BB receptor [26,27], and the FGF receptor [27], thereby attenuating ligand-induced downstream signaling. Besides its role in regulating growth factor-based signaling, PTP1B is also involved in regulating insulin- and leptin-induced signaling. As a result, several laboratories are actively developing PTP1B inhibitors that will interfere with its negative regulation of these important signaling processes [28-34].

Previous studies from this laboratory [35] demonstrated the expression of several PTPs, including, PTP1B, SHP-1, SHP-2, PTP-μ, and the dual-specificity phosphatase, PTEN, in rat corneal endothelial cells both in ex vivo corneas and in culture. Treatment of subconfluent rat corneal endothelial cells with the general phosphatase inhibitor, sodium orthovanadate (SOV), increased the relative number of cells entering the cell cycle, as indicated by positive staining for Ki67, a marker of actively cycling cells [36]. This result strongly suggested that phosphatase activity helps suppress cell cycle entry in corneal endothelium. Subsequent studies concentrated on the role of PTP1B in the regulation of EGFR signaling in rat corneal endothelium [37]. Western blot studies indicated that EGF induces phosphorylation of EGFR-Tyr992. Incubation of these cells with the PTP1B inhibitor, CinnGEL 2Me, both sustained EGF-induced phosphorylation of EGFR-Tyr992 and increased the number of cells entering the cell cycle, as indicated by immunostaining for Ki67. Together, these findings provide evidence that PTP1B plays a role in the negative regulation of EGFR signaling in rat corneal endothelial cells, at least at the level of Tyr992 phosphorylation.

The current studies move the inquiry from rat to human corneal endothelial cells. The goal of these studies was to determine whether PTP1B plays a role in negatively regulating cell cycle entry in EGF-stimulated HCEC. Studies were conducted to verify expression of PTP1B in human corneal endothelium both in ex vivo corneas and in culture, to compare the relative expression of PTP1B and EGFR in HCEC cultured from young and older donors, and to determine whether inhibition of the activity of PTP1B could increase cell cycle entry in HCEC in response to EGF stimulation.

## Methods

### Isolation and culture of human corneal endothelial cells

Donor human corneas were obtained through National Disease Research Interchange (NDRI, Philadelphia, PA). In accepting corneas from NDRI, the overall health of the donor before death was considered and tissue was rejected from donors with previous history or treatment that might damage the corneal endothelium, as indicated in the exclusion criteria that have been reported previously [6]. Handling of donor information by the source eye bank, NDRI, and this laboratory adhered to the tenets of the Declaration of Helsinki 1983 revision in protecting donor confidentiality. All corneas were preserved in Optisol-GS (Baush & Lomb, Rochester, NY) at 4 °C. Table 1 presents donor information for all corneas used in the current studies. For tissue culture studies, endothelial cells were isolated from donor corneas and cultured according to previously described protocols [4,5]. Briefly, Descemet's membrane with attached endothelium was dissected in small strips and then incubated in culture medium overnight at 37 °C to stabilize the cells. Culture medium consisted of OptiMEM-I (Invitrogen-Gibco, Grand Island, NY) supplemented with 8% fetal bovine serum (FBS; Hyclone, Logan, UT), 5 ng/ml epidermal growth factor (EGF; Upstate Biotechnologies, Lake Placid, NY), 20 ng/ml nerve growth factor (NGF; Biomedical Technologies, Stoughton, MA), 100 μg/ml bovine pituitary extract (Biomedical Technologies), 20 μg/ml ascorbic acid (Sigma-Aldrich, St. Louis, MO), 200 mg/ml calcium chloride, 0.08% chondroitin sulfate (Sigma-Aldrich), 50 μg/ml gentamicin (Invitrogen-Gibco), and antibiotic-antimycotic solution (Sigma-Aldrich) diluted 1:100. After gentle centrifugation, the medium was removed and 0.02% EDTA was added for 1 h at 37 °C to separate the cells. Following centrifugation, the isolated cells and pieces of Descemet's membrane still containing attached cells were resuspended in culture medium, plated in six-well tissue culture plates that had been pre-coated with FNC Coating Mix (Biological Research Faculty & Facility, Inc., Ijamsville, MD), and incubated at 37 °C in a 5% CO_2_ humidified atmosphere. Medium was changed every-other day. After cells reached confluence, they were subcultured at a 1:2 ratio. HCEC at passage 2 were used for all experiments.

**Table 1:** Donor information.

**Experiment**	**Age**	**Time**	**Days**	**Cause of Death**
PTP1B ICC	16	17:00	2	Multiple trauma
	18	8:55	6	Sledding accident
	55	5:45	2	Lung cancer
	59	12:04	3	Traumatic injury
	61	2:26	4	Cerebrovascular accident
	66	7:50	6	Gunshot wound

Western Blots: EGFR/PTP1B	NB*	6:56	4	Cardiac arrest
	NB	2:50	7	Anoxic brain injury
	2	11:02	6	Smoke inhalation
	3	12:12	6	Thermal burns
	60	5:38	4	Thermal burns
	66	11:38	2	Cardiac arrest
	74	6:50	3	Cancer
	78	7:27	3	Cardiac arrest

BrdU Assay	16	19:55	3	Motor vehicle accident
	18	12:01	6	Motor vehicle accident
	72	8:06	1	Acute cardiac event
	72	9:07	1	Myocardial Infarction

Time; Time from death to preservation (h). Days; Days from death to culture or fixation. The single asterisk indicates newborn and the double asterisk indicates the same donor source as used for the western blot verification of PTP1B expression.

### Immunocytochemical localization of PTP1B in ex vivo corneas and cultured HCEC

Table 1 provides information regarding the donor corneas used for these studies. Corneas from four different donors were used to localize PTP1B in ex vivo corneal endothelium, while corneas from two different donors were used as a source of cells for culture. Corneas were washed three times with culture medium and incubated overnight (37 °C, 5% CO_2_) to stabilize the cells. After incubation, the tissues were washed with phosphate-buffered saline (PBS; Invitrogen) and fixed for 10 min in 100% methanol at −20 °C. All further incubations were at room temperature. Corneas were cut in quarters, washed in PBS three times for 10 min each, then permeabilized for 10 min in PBS containing 1% Triton X-100 (Sigma-Aldrich). Before they were stained with antibodies, tissues were washed with 0.1% Triton X-100 in PBS and incubated for 10 min in blocking buffer containing 0.1% Triton X-100 and 4% BSA in PBS. After a 2 h incubation in mouse-anti-PTP1B (PH01; Calbiochem) diluted 1:50 in 0.1% Triton X-100 in PBS, the tissues were rinsed with 0.1% Triton X-100 in PBS and reincubated for 10 min in blocking buffer. Corneas were then incubated for 1 h in FITC-conjugated donkey anti-mouse IgG (Jackson ImmunoResearch), diluted 1:50 with 0.1% Triton X-100 in PBS. Tissue incubated in secondary antibody alone acted as a negative control. Each corneal quarter was mounted endothelial-side up on a glass slide. Coverslips were mounted in medium containing propidium iodide (PI; Vector Laboratories, Burlingame, CA) to stain all nuclei. Digital images were obtained using a Leica TSC-SP2 confocal microscope (Bannockburn, IL). A Z-series through the tissue was captured with a step size of 0.5 μm per image. Z-series images were collapsed onto a single image plane by projecting the maximal pixel intensity of the images. For immunolocalization of PTP1B in cultured cells, Passage-2 HCEC were grown to approximately 50% confluence. HCEC were then washed, fixed, and processed for immunostaining of PTP1B as described for the ex vivo corneas. Cells incubated in secondary antibody alone acted as negative controls. Staining of cultured cells was visualized using an Eclipse E800 Nikon Microscope with VFM Epi-Fluorescence Attachment (Nikon, Inc., Melville, NY) equipped with a Spot digital camera and Spot Advanced version 4.5 CE software (Diagnostic Instruments, Sterling Heights, MI).

### Western blot verification of PTP1B expression in HCEC

HCEC were cultured from a newborn and a 74-year-old donor as indicated in Table 1. Protein was extracted from confluent Passage-2 cells by incubating cells for 30 min at 4 °C in lysis buffer containing 1% Triton X-100, 250 mM NaCl, 2 mM EDTA, 50 mM Tris-HCl (pH 7.4), 10 μg/ml aprotinin, 10 μg/ml leupeptin, 1 mM phenylmethylsulfonyl fluoride, 50 mM sodium fluoride, and 0.1 mM sodium orthovanadate (all from Sigma-Aldrich), followed by homogenization and centrifugation. The protein concentration of the supernatants was determined spectrophotometrically. Equal concentrations of soluble protein were loaded on 10% Bis-Tris gels (Invitrogen) for SDS–PAGE and then electrophoretically transferred to a polyvinylidene difluoride (PVDF) membrane (Millipore, Bedford, MA). A commercially prepared whole cell lysate from SW480 cells, a human colonic adenocarcinoma cell line (Santa Cruz Biotechnology Inc., Santa Cruz, CA), acted as a positive control for PTP1B. Non-specific binding was blocked by incubation of the membranes for 1 h at room temperature in 5% non-fat dry milk and 0.1% Triton X-100 in PBS. Membranes were then incubated overnight at 4 °C with 1.25 μg/ml mouse anti-PTP1B (PH01, as above). Blots were rinsed three times for 10 min each with 0.1% Triton X-100 in PBS and then incubated 1 h at room temperature with HRP-conjugated donkey anti-mouse IgG, diluted 1:5000. Primary and secondary antibodies were diluted with 5% non-fat dry milk, 0.1% Triton X-100 in PBS. Membranes were washed three times 10 min with 0.1% Triton X-100 in PBS, and antibody binding was visualized using a chemiluminescent substrate (SuperSignal West Pico; Pierce, Rockford, IL).

### Western blot analysis of EGFR and PTP1B expression in HCEC from young and older donors

HCEC were cultured from four young and four older donors as indicated in Table 1. Note that one sample from a newborn donor and the sample from the 74-year old donor were also used for western blot verification of PTP1B as described above. Protein was extracted from passage 2 cells as above and supernatants were stored at −80 °C until used for analysis. Polyacrylamide gel electrophoresis and electrophoretic transfer of peptides to PVDF membranes were performed as previously described. Whole cell lysate from SW480 cells acted as a positive control for PTP1B, while protein from EGF-stimulated A431 cells (Santa Cruz) acted as a positive control for EGFR. Non-specific binding was blocked by incubation for 1 h at room temperature in 5% non-fat dry milk and 0.1% Triton X-100 in PBS. Membranes were incubated overnight at 4 °C with either 1.25 μg/ml mouse anti-PTP1B (PH01, as above) or rabbit anti-EGF receptor (1:1,000; Cell Signaling Technology Inc., Danvers, MA). Blots were rinsed and then incubated 1 h at room temperature with either HRP-conjugated donkey anti-mouse IgG, diluted 1:5000 to detect PTP1B, or HRP-conjugated donkey anti-rabbit IgG, diluted 1:2,000 to detect EGFR. Membranes were washed three times 10 min with 0.1% Triton X-100 in PBS, and antibody binding was visualized using a chemiluminescent substrate (SuperSignal West Pico; Pierce, Rockford, IL). To prepare controls for protein loading, bound antibodies were stripped from membranes by incubation for 15 min at room temperature in buffer containing 2% SDS, 62.5 mM Tris-HCl (pH 6.8) and 100 mM 2-mercaptoethanol (all from Sigma) and reprobed with 1 μg/ml mouse anti-β-actin (Sigma) diluted in 5% non-fat dry milk, 0.1% TritonX-100 in PBS followed by incubation in HRP-conjugated donkey anti-mouse IgG diluted 1:10,000. Studies were conducted in duplicate. Semi-quantitative analysis was performed by densitometry using an image-analysis software program (NIH Image-J 1.38; National Institutes of Health, Bethesda, MD). Protein expression was represented as a value relative to that of β-actin. Statistical analysis was performed using unpaired Student's *t*-test. A p<0.05 was considered statistically significant.

### BrdU incorporation following EGF stimulation (+/−) PTP1B inhibitor

HCEC cultured from four donors were used for these studies (see Table 1). To wean confluent passage 1 cells from the 8% FBS and growth factors present in the normal culture medium, cells were incubated for four days before subculture in medium containing 4% FBS without additional growth factors or pituitary extract. HCEC were then trypsinized, seeded into four-well chamber slides (Nalge Nunc International, Naperville, IL) pre-coated with FNC Coating Mix, and incubated for 48 h in medium containing 0.1% FBS without added growth factors or bovine pituitary extract. This concentration of FBS was tested previously and found to maintain the health of cultured HCEC without inducing proliferation (data not shown). Cells in one-half of the wells were pre-incubated for 1 h in the same medium plus 25 μM CinnGEL 2Me (BIOMOL Research Laboratories Inc., Plymouth Meeting, PA), a novel peptide inhibitor of PTP1B [38]. CinnGEL 2Me was prepared by reconstitution in dimethyl sulfoxide (DMSO, Sigma-Aldrich) according to the supplier's instructions. Control cells were pre-incubated for 1 h in culture medium plus the same volume of DMSO. EGF (25 ng/ml), plus 0.5 μl of Cell Proliferation Labeling Reagent containing BrdU (Amersham Biosciences, Buckinghamshire England), was then added to all culture wells. At 0, 12, 24, and 48 h after EGF stimulation, cells were fixed with 100% methanol at −20 °C. Fixation, blocking and antibody incubation steps were the same as those described above for immunocytochemical localization of PTP1B. Mouse anti-BrdU was used as primary antibody and FITC-conjugated donkey anti-mouse IgG (diluted 1:200) was used as secondary antibody. Secondary antibody alone acted as a negative control. Coverslips were mounted in medium containing PI to stain all nuclei. Positive staining of cultured cells was visualized using the Nikon Eclipse E800 microscope described above. Duplicates were prepared for each time point and condition. Seven images were taken per culture well with a 40X objective lens. NIH Image-J 1.38 was used to count total PI-stained nuclei and total BrdU-positive nuclei. The relative percent of BrdU incorporation was calculated for each donor and time point. The number of BrdU-positive nuclei was then averaged for each time point. Differences in the counts were analyzed with Student's unpaired *t*-test. A p<0.05 was considered significant.

## Results

### PTP1B localization in ex vivo human corneal endothelium and in cultured HCEC

**Figure 1:**
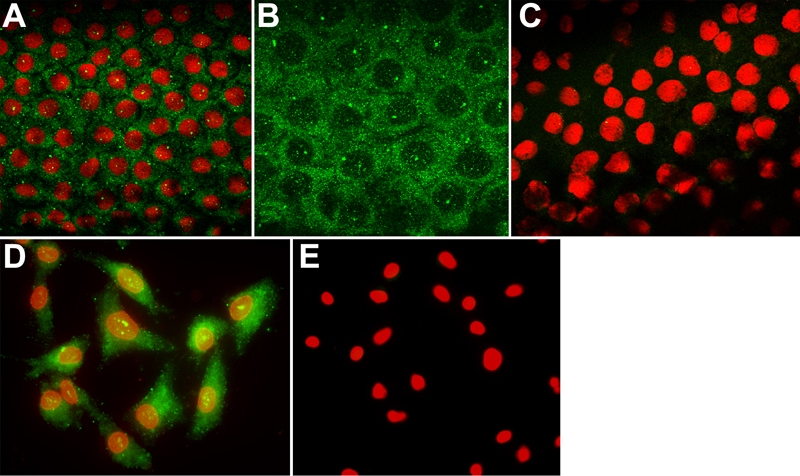
Representative images of PTP1B localization in ex vivo human corneal endothelium and in cultured HCEC. **A**: Representative image of PTP1B (green) staining in the endothelium of a cornea obtained from a 59-year-old donor. PTP1B is localized in a punctate pattern mainly within the cytoplasm. Some punctate staining is also visible in nuclei (red). Note that larger dots of intense stain can be observed scattered within the tissue or on individual cells. This appears to be due to non-specific antibody deposition, although all antibodies were centrifuged at high-speed before use. **B**: Higher magnification image of HCEC in ex vivo corneal tissue showing more detail of the PTP1B localization pattern. **C**: Negative control of ex vivo tissue in which corneas were incubated with secondary antibody alone. Image is an overlay from both the FITC and rhodamine channels. **D**: PTP1B staining in subconfluent HCEC cultured from a 55-year-old donor results in a similar punctate pattern in the cytoplasm and nucleus. **E**: Negative control in which cells were incubated with secondary antibody only. Image is an overlay from both the FITC and rhodamine channels. green: PTP1B. red: Propidium iodide. Original magnification for **A**, **C**, **D**, and **E**: 40X. Original magnification for **B**: 100X.

Corneas from donors aged 18, 59, 61, and 66 years old were used for localization of PTP1B in the endothelium in situ. Corneas from donors aged 16 and 55 years old were sources of cultured HCEC. Positive staining for PTP1B demonstrated the expression of this important phosphatase in HCEC. Representative images of PTP1B immunostaining are presented in Figure 1. A similar pattern of PTP1B staining was found in HCEC both in situ (Figure 1A,B) and in culture (Figure 1D). Intense punctate staining for PTP1B was located within the cytoplasm, while some punctate staining was also observed in nuclei. No staining was observed in controls incubated in secondary antibody alone (Figure 1C,E). Comparison of the images shows no obvious age-related difference in the relative localization pattern of HCEC either in situ or in culture.

### Verification of PTP1B protein expression in HCEC

**Figure 2:**
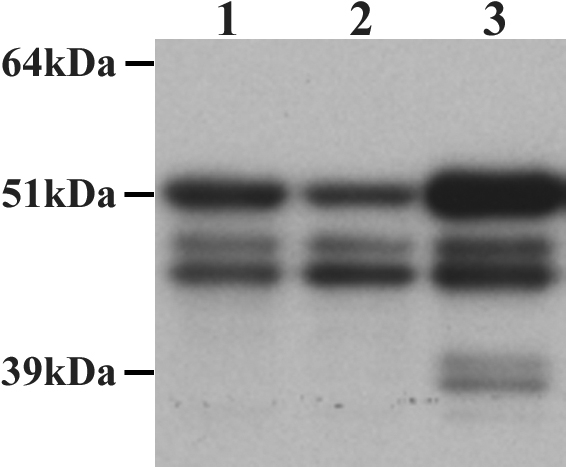
Western blot detection of PTP1B protein bands in HCEC. Confluent Passage-2 HCEC were cultured from a newborn (lane 1) and a 74-year-old donor (lane 2) and processed for western blot detection of PTP1B. A commercially prepared whole cell lysate of SW480 cells was used as a positive control (lane 3). Blots showed that the two HCEC samples yielded a band at approximately 50 kDa, indicating the presence of full-length PTP1B, as well as two additional bands of approximately 48 kDa and 46 kDa, possibly representing truncated forms. The same three bands were observed in the SW480 positive control sample, as well as lower molecular weight bands around 39–37 kDa that may result from non-specific proteolytic cleavage. Note the variability in density of the three PTP1B-positive bands within the two HCEC samples.

Western blot studies were conducted to verify PTP1B protein expression in HCEC. Table 1 presents information regarding the two donors used for this study. As seen in Figure 2, samples extracted from passage 2 HCEC (lanes 1 and 2) yielded three PTP1B-positive bands-a band at 50 kDa, as well as two additional bands migrating at approximately 48 kDa and 46 kDa. The SW480 cell positive control (lane 3) yielded a total of five bands-three corresponding to the same bands observed in the HCEC samples and two additional lower molecular weight bands at approximately 39 kDa and 37 kDa. Published studies of PTP1B protein expression in several different cell types, including HeLa cells, human diploid fibroblasts, CV-1 cells, and COS-1 cells, indicate that gel electrophoresis of full-length PTP1B yields a band with a relative molecular weight of approximately 50 kDa [18,20,21]. This strongly suggests that the 50 kDa band found on the blots of HCEC corresponds to full-length PTP1B. PTP1B has also been detected at somewhat lower molecular weights. For example, bands of approximately 48 kDa [39] and 46 kDa [21] have been observed and appear to represent truncated forms of PTP1B that are missing the COOH-terminal hydrophobic domain [21]. Thus, the 48 kDa and 46 kDa bands found in all three samples in the current study appear to represent truncated forms of PTP1B. Non-specific proteolytic cleavage of PTP1B can yield a band migrating at approximately 37 kDa [17,20,21]. Two bands migrating around 39 kDa and 37 kDa were visible in the commercially prepared lysate of SW480 cells, suggesting that a portion of PTP1B was nonspecifically cleaved in this sample. These lower molecular weight bands were not detected in the samples prepared from HCEC. Together, these results verified the expression of PTP1B in HCEC and provided suggestive evidence that this protein tyrosine phosphatase exists in multiple forms within the cell. Differences in the relative densities of the three PTP1B bands were also noted. For example, in the HCEC sample in lane 1, the 50 kDa band density appeared to be higher than that of either the 48 kDa or 46 kDa bands, whereas, in the sample in lane 2, the 46 kDa band appeared denser than the 50 kDa band. The fact that intra-sample differences were observed in this study was important, because it affected how total PTP1B expression was calculated in the comparative western blot studies described below. In addition, the protein samples used for this study were obtained from a newborn and a 74-year-old donor. It is possible that the observed intra- and/or inter-sample differences in relative density of the three PTP1B-positive bands were due to age-related alterations in PTP1B expression. This possibility was further explored in the comparative studies described below.

### Relative PTP1B and EGF receptor protein expression in HCEC from young and older donors

**Figure 3:**
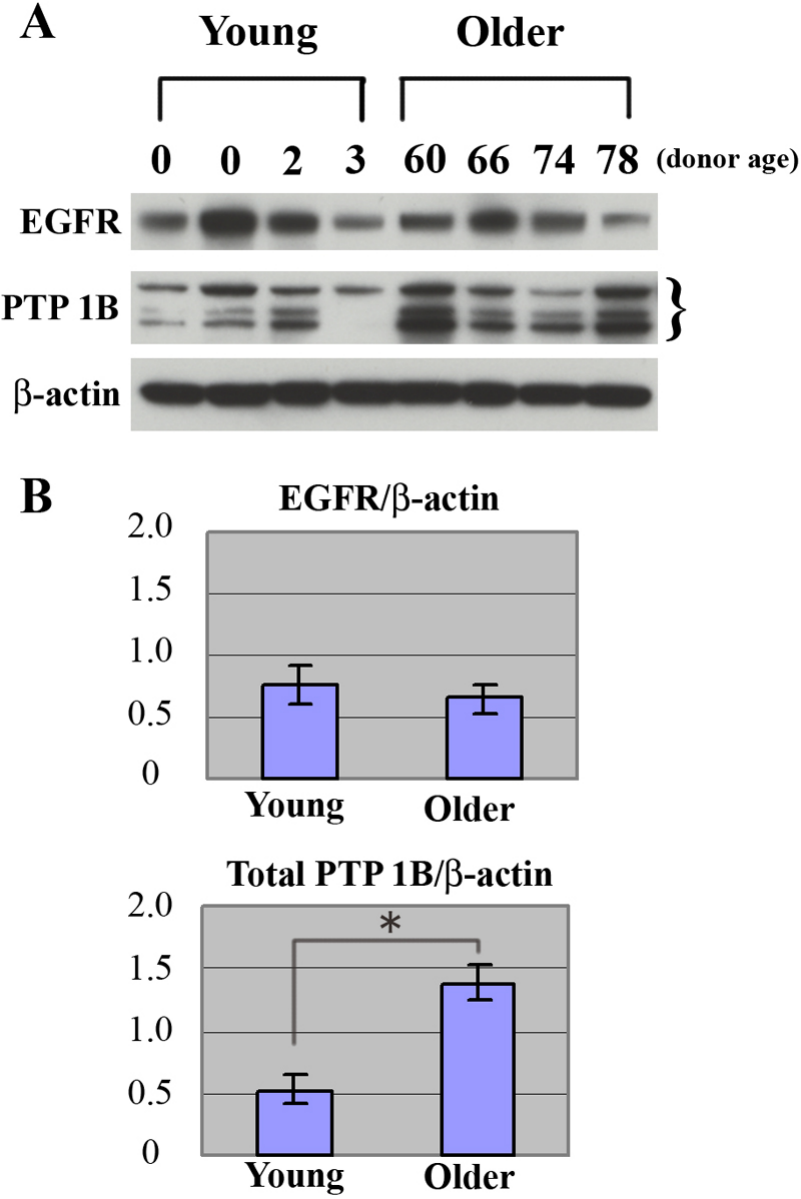
Comparison of EGFR and PTP1B protein expression in HCEC cultured from 4 young and 4 older donors. **A**: Western blots demonstrate the relative expression of EGFR and PTP1B in each of the 8 donor samples. EGFR was expressed in all 8 samples, although the band density varied somewhat among the samples. Three PTP1B bands were observed in most samples. These bands are indicated within brackets. Blots for the positive controls for EGFR (EGF-treated A431cells) and PTP1B (SW480 cells) are not shown, but bands were obtained at the same relative molecular weights as in the HCEC samples. β-Actin was used as a loading control for both EGFR and PTP1B. **B**: Comparison of the average band density for EGFR and PTP1B in samples from young and older donors. Bars represent SEM. Asterisk indicates statistical significance (p=0.024).

Previous studies from this laboratory demonstrated that HCEC are able to proliferate in response to EGF, but that HCEC cultured from older donors (>50 years old) are less responsive than cells from young donors (<30 years old) [5,6]. It is possible that, in HCEC from older donors, the relative expression of EGF receptors might be reduced, leading to a weaker response to EGF. Since PTP1B is known to dephosphorylate activated EGFRs, it is also possible that there may be an age-related difference in the relative expression of PTP1B in HCEC from older donors that would underlie the observed difference in response to EGF. Western blot studies were therefore conducted to compare the relative protein expression of EGFR and PTP1B in HCEC. Protein samples were prepared from confluent passage 2 cells cultured from four young (two newborns, 2-year old and 3-year old) and four older donors (aged 60, 66, 74, and 78 years old). Table 1 provides more information and indicates that one newborn donor sample and the sample from the 74-year-old donor used in these studies were also used for the western blot study described above. Results are presented in Figure 3. Figure 3A shows that EGFR protein was detected in all HCEC samples. A band of the same relative molecular weight was detected in EGF-stimulated A431 cells, which acted as a positive control for the western blot (data not shown). The relative band density of EGFR tended to vary from donor-to-donor; however, when band densities were normalized to β-actin and average band densities compared, there was no statistically significant difference (p=0.444>0.05) in average EGFR expression between the two age-groups (Figure 3B). As initially observed in the western blot study discussed above and shown in the expanded comparative study in Figure 3A, the relative density of each of the three PTP1B-positive bands tended to vary from sample to sample; however, there was no consistent, age-related intra-sample density pattern observed. To calculate total PTP1B expression in each donor sample, the density of all three bands was added together and the total density was then normalized to β-actin. The total density for PTP1B in each sample was then used to calculate the average total density of PTP1B within the two age-groups. As shown in Figure 3B, the average total density of PTP1B was significantly higher (p=0.024<0.05) in HCEC samples from older donors compared with young donors, indicating that PTP1B is expressed at higher levels in HCEC from older donors.

### PTP1B inhibitor increases BrdU incorporation following EGF stimulation

**Figure 4:**
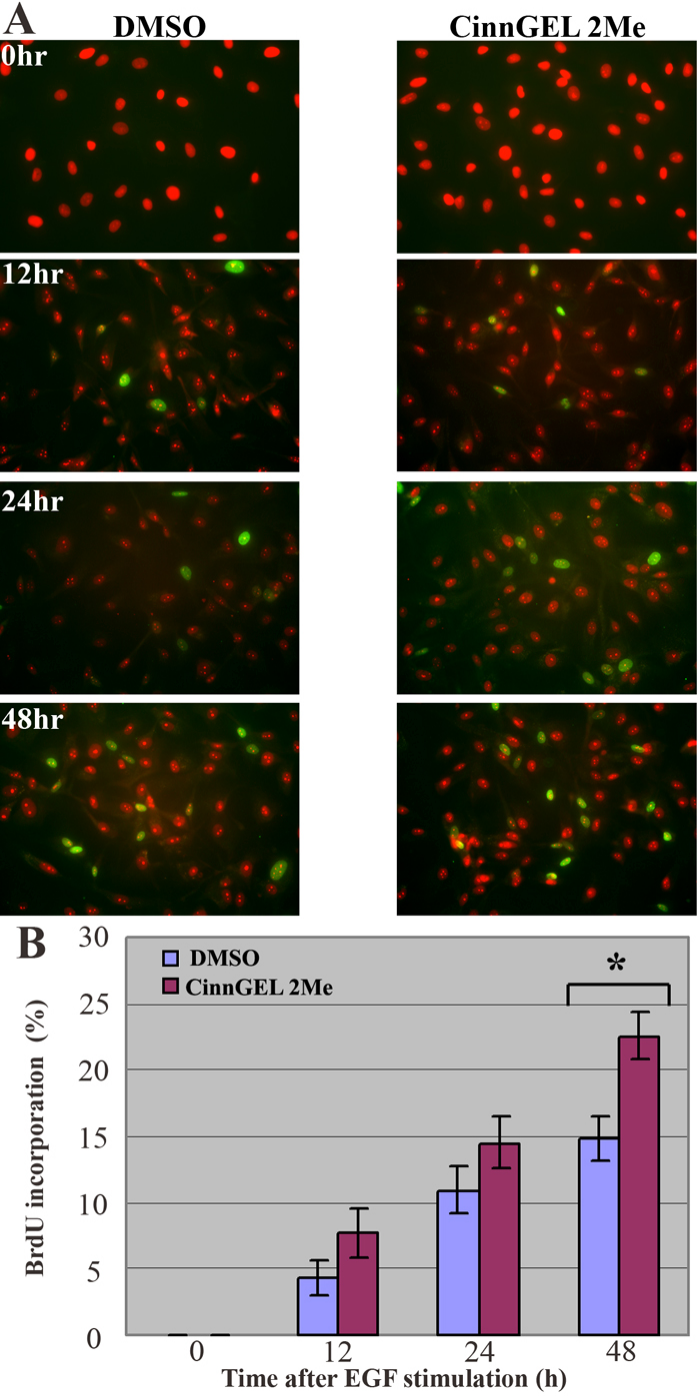
Effect of PTP1B inhibitor on cell cycle entry in response to EGF stimulation. Subconfluent HCEC were pre-incubated for 1 h in medium containing either DMSO alone or 25 μM of the PTP1B inhibitor, CinnGEL 2Me, diluted in DMSO. Following this pre-incubation step, 25 ng/ml EGF was added to all cultures. Samples were taken for BrdU staining at 0, 12, 24, or 48 h after EGF addition. **A**: Representative images showing BrdU staining (green) in subconfluent HCEC from a 72 year-old donor incubated in either DMSO alone or in CinnGEL 2Me PTP1B inhibitor. Cultures were counterstained with propidium iodide (PI) to reveal all nuclei (red). Final magnification: 400X. **B**: Bar graph shows the average percent of BrdU-positive HCEC at each time point. Bars represent SEM. The asterisk indicates statistical significance at p=0.019.

Experiments were then conducted to determine whether inhibition of PTP1B activity could increase S-phase entry in HCEC. The inhibitor chosen for this study was CinnGEL 2Me. The free cinnamic acid component of CinnGEL 2Me inhibits PTP1B (IC_50_=1.3 μM), while the methyl ester is added for cell permeability. According to the supplier, CinnGEL 2Me is hydrolyzed to active inhibitor by intracellular esterases. In studies of rat corneal endothelial cells [37], the effect of CinnGEL 2Me was tested at a final concentration of 1 mM and found to increase cell cycle entry as indicated by quantification of Ki67 staining. Prior to this study in HCEC, a range of CinnGEL 2Me concentrations was tested both for its effect on S-phase entry and on cell viability. A final concentration of 25 μM was found to be optimal and was used for the subsequent studies. HCEC were cultured from donors aged 16 and 18 and two different 72-year-old donors (see Table 1). Subconfluent Passage-2 cells were pretreated for 1 h with 25 μM CinnGEL 2Me. Since CinnGEL 2Me requires reconstitution in DMSO, control cells were pre-treated for 1 h with the same concentration of DMSO minus the inhibitor. Cells were then stimulated with 25 ng/ml EGF and stained at 0, 12, 24, and 48 h for BrdU incorporation to test the effect of PTP1B inhibition on S-phase entryE cultured. For these studies, BrdU was used to specifically indicate S-phase entry. Figure 4A presents a representative example of BrdU staining in HCEC from one of the 72 year-old donors. No BrdU staining was observed at the 0 time point or in control tissue incubated in secondary antibody only (data not shown). Images show a gradual increase in the relative number of BrdU-stained cells. Figure 4B shows that, under both conditions, the average number of BrdU-positive cells increased in a time-dependent manner. At the 12, 24, and 48 h time points, the average BrdU incorporation tended to be higher in cells treated with the PTP1B inhibitor. By 48 h after EGF stimulation, BrdU incorporation was significantly higher (p=0.019<0.05) in CinnGEL 2Me-treated cells.

## Discussion

Previous studies documented the expression of PTP1B in rat corneal endothelial cells [35,37]; however, the current studies are the first to demonstrate expression of this important PTP in HCEC. Results indicate that PTP1B is expressed in HCEC and that the pattern of PTP1B localization is similar in ex vivo endothelium and in cultured cells, indicating that no gross change in PTP1B location occurs upon culturing. The punctate cytoplasmic pattern observed in HCEC is consistent with that observed in ex vivo rat corneal endothelium [35]. A similar pattern has been demonstrated in several cell types [21,22,39-41] and suggests that, in HCEC, PTP1B is mainly localized to the cytoplasmic surface of the endoplasmic reticulum. Some punctate staining was also observed in the nuclei of HCEC both in situ and in culture. This finding is consistent with studies in other cell types [21,23], which have shown that at least a subset of PTP1B may be associated with the nuclear membrane. Co-localization of PTP1B with specific membrane markers will be needed to verify its specific subcellular localization. PTP1B is implicated in dephosphorylation and inactivation of several plasma membrane-associated RTKS, including EGFR [24-26]. Immunolocalization studies in ex vivo rat corneal endothelium [37] demonstrated a time-dependent internalization of EGFR at cell borders following EGF stimulation, suggesting that EGFR could interact with and be regulated by PTP1B within the cell. Similar studies are needed to verify that this interaction also occurs in HCEC. It will also be important to clearly demonstrate that PTP1B is directly involved in time-dependent dephosphorylation of EGFR following EGF stimulation of HCEC.

Protein extracts of HCEC, as well as the commercial SW480 cell lysate that was used as a positive control, consistently showed the presence of three bands for PTP1B on western blots-one band at 50kDa (the relative molecular weight expected for full-length PTP1B) and two bands of approximately 48 kDa and 46 kDa. As discussed above, these two lower molecular weight bands most likely correspond to truncated forms of PTP1B. The exact nature and function of these truncated forms is unclear and needs to be further investigated. The bands identified on the blots of HCEC and SW480 cells do not appear to correspond to PTP1B forms described in HeLa cell extracts by Schievella, et al. [42]. Those studies identified a single PTP1B band migrating more slowly than the 50 kDa band in samples from cells synchronized in G_2_/M-phase with nocodazole. This slower migrating form was identified as a serine-phosphorylated (“mitotic”) form of PTP1B. In the current studies, the position of the three PTP1B bands in HCEC relative to that of the commercial molecular weight markers and to the bands of the positive control indicates that the slowest migrating band in the HCEC samples corresponds to the full-length 50kDa form of PTP1B and not to a slower migrating “mitotic” form. In addition, fully confluent cells were used for these studies and the presence of mitotic cells in the cultures is highly unlikely. Interestingly, the relative density of the three bands differed among the HCEC samples. The specific nature of the two lower molecular weight forms and the reason why their relative concentrations differ in HCEC remains to be elucidated.

Previous studies from this laboratory demonstrated an age-related decrease in the responsiveness of HCEC to EGF stimulation [5,6]; however, the molecular basis for this decrease is not known. One possibility is that the expression of EGFR decreases in an age-related manner; however, results of the current western blot studies demonstrate that the relative expression of EGFR protein did not differ significantly with donor age. This finding differs from that of Lopez, et al. [43], who used flow cytometry to quantify EGF receptors in corneal cells. In that study, Passage-7 HCEC cultured from an infant donor, which exhibited senescence characteristics, expressed a considerably higher number of EGF receptors (>30,000/cell) than early passage cells from an infant donor (5,142 ±2,412/cell). This discrepancy in results could be due to differences exhibited by cells aged in culture through multiple passages versus early-passage cells cultured from young and older donors or to the detection methods used for assay. Results similar to those in the current study have been reported in other cell types. For example, EGF-stimulated DNA synthesis was found to be decreased in primary hepatocytes cultured from 24 month-old rats compared to cells cultured from 6 month-old rats, although no age-related difference in EGFR density or binding affinity was detected [44]. Similarly, decreased DNA synthesis was observed in human diploid fibroblasts aged by passage in culture without a concomitant change in EGFR expression [45,46]. Together, these results suggest that the difference in responsiveness to EGF exhibited by aged cells may not be specifically due to differences in EGFR expression, but to decreased downstream signaling [45]. This idea is supported by studies in mouse [47] and rat hepatocytes [48], which found that decreased levels of DNA synthesis in old mice were associated with decreased activation of ERK and decreased tyrosine phosphorylation of EGFR. Studies of in vitro aged human dermal fibroblasts by Tran, et al. [49] demonstrated that EGF receptor autophosphorylation was significantly decreased upon EGF stimulation, as was phosphorylation of the downstream mediator, Shc. These age-related changes were paralleled by increased dephosphorylation of EGFR, increased PTPase activity, and increased expression of the protein tyrosine phosphatases, PTP1B and SHP-1. These findings provide evidence that, with age, cells become less sensitive to EGF stimulation due, at least in part, to increased dephosphorylation of EGFR by PTPs, including PTP1B, resulting in decreased downstream signaling. Interestingly, western blots of HCEC showed no significant age-related change in EGFR expression, but did detect a statistically significant increase in PTP1B expression in HCEC cultured from older donors. Therefore, it is possible that the difference in responsiveness to EGF exhibited by HCEC from older donors is not due to differences in EGFR expression but to decreased downstream signaling resulting from increased PTP1B-induced EGFR dephosphorylation. This intriguing hypothesis requires further testing.

To test whether PTP1B plays a role in the negative regulation of EGF signaling in HCEC, we employed the commercially available PTP1B inhibitor, CinnGEL 2Me. In previous studies, treatment of rat corneal endothelial cells with this inhibitor before EGF stimulation resulted in an increase in Ki67-positive cells [37]. Ki67 is a recognized marker of actively cycling cells, because its expression is detectable in late G_1_-phase through mitosis, but not in quiescent cells [37,50]. In the current studies, we chose to use BrdU to more specifically mark entry into S-phase of the cell cycle. Although EGF stimulated S-phase entry both in the presence and absence of the PTP1B inhibitor, inhibition of PTP1B resulted in a significant increase in S-phase cells by 48 h after EGF addition, strongly suggesting that PTP1B negatively regulates EGF-stimulated proliferation. It should be noted that the population doubling time (PD) of HCEC is quite slow. The average PD for HCEC cultured from young donors is 46.25 h, while the PD for cells from older donors is 90.25 [6]. Thus, it was not unexpected that these results only showed a statistical significance 48 h after EGF stimulation. Additional studies are needed to determine whether PTP1B inhibition will increase division of HCEC, particularly from older donors.

In summary, PTP1B is expressed in HCEC, as well as in rat corneal endothelial cells. Staining patterns strongly suggest that at least a subset of PTP1B is localized to the cytoplasm and most likely to the endoplasmic reticulum-a site at which PTP1B is known to dephosphorylate EGFR in other cells. Interestingly, PTP1B expression, but not EGFR expression was elevated in HCEC from older donors. It is possible that the reduced sensitivity of these cells to EGF stimulation is due, at least in part, to increased PTP1B activity, resulting in decreased downstream signaling and a reduced number of cells entering the cell cycle. These findings are relevant to understanding the molecular basis for the decreased proliferative response of HCEC to EGF (and possibly to other RTKs) and may lead to treatments to overcome decreased downstream signaling by inhibiting PTP1B, thereby increasing proliferation and endothelial cell density.
